# Colonic Lipoma: A Rare Cause of Intussusception

**DOI:** 10.7759/cureus.48074

**Published:** 2023-10-31

**Authors:** Manjeet K Goyal, Yogesh K Gupta, Varun Mehta, Arshdeep Singh, Ajit Sood

**Affiliations:** 1 Gastroenterology and Hepatology, Dayanand Medical College and Hospital, Ludhiana, IND; 2 Gastroenterology, Dayanand Medical College and Hospital, Ludhiana, IND

**Keywords:** adult onset, including constipation, constipation misperception, colonic lipoma, ileocolic intussusception

## Abstract

The most common and challenging chief complaint in the emergency department is abdominal pain. Intussusception, although rare in adults, is an important etiology to consider. The diagnosis is often delayed because of the nonspecific symptoms, especially in adults. This case highlights a rare case of intussusception in a middle-aged male with a colonic lipoma as a leading point. Endo-loop was applied to the colonic lipoma, leading to the resolution of intussusception. Therefore, this can be an effective alternative to surgery in select cases.

## Introduction

Intussusception is a significant medical entity characterized by the invagination of the proximal segment of the gastrointestinal tract into the lumen of the neighboring segments [1]. Intestinal obstruction is a critical medical condition characterized by the blockage of the gastrointestinal tract, which can result in life-threatening consequences if left untreated. Intussusception is commonly observed in pediatric patients and manifests with the characteristic triad of cramping abdominal pain, bloody diarrhea, and the presence of a palpable, sensitive mass. Bowel intussusception in adults, on the other hand, is an uncommon disorder, accounting for 5% of all intussusception cases and 1% to 5% of bowel obstructions in the adult population [1]. Intussusception as a result of colonic lipoma is an exceptionally infrequent phenomenon. Colonic lipomas are rare kinds of benign, non-epithelial neoplasms composed of adipose tissue [2]. The majority of colonic lipomas are often asymptomatic and are often discovered accidentally through procedures such as colonoscopy, surgery, or postmortem examinations. However, it is worth noting that around 25% of individuals may experience the onset of symptoms such as stomach discomfort, diarrhea, constipation, the resemblance to colon cancer, and rarely, intussusception [2,3].

In this report, we provide a clinical case involving a male individual of middle age who received a diagnosis of colo-colonic intussusception, with a leading point identified as an ascending colon lipoma. The lipoma was encircled using an endoloop through an endoscopic procedure, and the patient was placed under postoperative observation. After a period of eight weeks, both the lipoma and intussusception were resolved, with the latter resolving immediately after the procedure. Hence, our case exhibited distinctive characteristics in terms of the primary focus of interest. Furthermore, it involved the use of an endo-loop using an endoscopic approach, which served as an alternative to the conventional surgical intervention often employed as the ultimate therapy. This case report demonstrates that endoscopic intervention can serve as a viable therapeutic option for select intussusception patients.

## Case presentation

**Figure 1:**
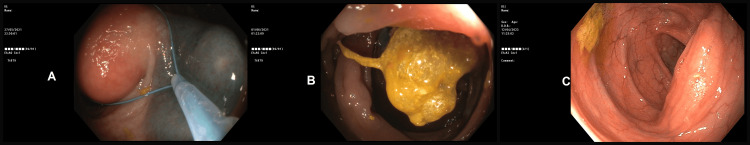
Endoscopic view A: Endoloop was applied to a sub-mucosal mass of 4 cm in ascending colon with a 'pillow sign', which is likely a lipoma; B: Mass was cauterized to reveal its contents; C: Resolution of mass after eight weeks

A 55-year-old male presented with severe, diffuse, and non-radiating abdominal pain for three days, with no association with food intake and no aggravating or relieving factors. He had a past history of chronic constipation with no alarming symptoms (weight loss, anorexia, blood in stools, etc.). He was taking laxatives daily. On physical examination, there was tachycardia (heart rate 112/min). He had a respiratory rate of 18/min, a blood pressure of 110/70 mmHg, a soft abdomen that was mildly tender to palpation, and positive bowel sounds. Initial laboratory tests were within normal limits; the workup was non-significant with a normal white cell count. A CT of the abdomen was suggestive of colo-colonic intussusception with ascending colon lipoma as the leading point. A surgical consult was obtained, and a colon resection was recommended. The patient refused to undergo the procedure. The decision to use air insufflation was then made to un-telescope the intussusception and relieve the obstruction. On further investigations, a colonoscopy was done, which revealed a 4 cm sub-mucosal mass with a 'pillow sign' being positive, most likely lipoma (Figure 1) as the leading point of intussusception. After informed consent and under general anesthesia, an endoloop was applied around the lipoma and was vigilantly monitored thereafter. He was discharged seven days after the procedure in stable condition with a resolution of symptoms. Eight weeks later, the colonoscopy was suggestive of the resolution of lipoma and intussusception as well. The patient is currently doing well with follow-up.

## Discussion

The invagination of an intestinal loop with a mesenteric fold (intussusceptum) in the lumen of a continuous segment of the intestine after peristalsis is known as intestinal intussusception [3]. Intussusception is a condition that is prevalent among children but relatively rare among adults. Approximately 95% of cases of intussusception are observed in pediatric patients, while the remaining 5% occur in adults, with pathological neoplasms serving as the primary cause. The condition has the potential to result in blockage of the inner passage, leading to further involvement of the mesentery with compromised blood flow, ultimately resulting in tissue damage and cell death. The condition has the potential to result in a range of consequences, including but not limited to intestinal blockage, gut necrosis, and sepsis.

Colonic lipomas with a size less than 2 cm often do not exhibit symptoms; however, those above 4 cm are symptomatic in the majority of cases. These symptomatic lipomas manifest with non-specific symptoms including abdominal discomfort, perforation, constipation, blockage, and bleeding [4]. The clinical manifestation of intussusception in adult individuals is characterized by non-specific symptoms, with the majority of cases exhibiting chronic or subacute indications that are indicative of chronic constipation [4]. The prevalence of the illness process is higher in the pediatric population in comparison to adults. However, the presence of this condition in children is often attributed to a pathological lead point, such as a tumor [5-7].

The diagnosis of intussusception in adults is a challenging task that necessitates a heightened level of clinical suspicion. Typically, colonic lipomas are asymptomatic and are commonly identified as accidental findings during procedures such as colonoscopy, surgery, and autopsy [8,9]. The occurrence of intussusception in adult patients is frequently accompanied by pain, which is reported as the most prevalent symptom, with a prevalence ranging from 71% to 90% of patients. The intermittent nature of this discomfort often leads to a delayed diagnosis. Computed tomography continues to be the preferred diagnostic modality for intussusception due to its ability to accurately identify the intussusceptive tissue and the lead point [10]. Nevertheless, the identification of the underlying etiology of intussusception by CT imaging continues to pose a significant difficulty. Colonic lipomas manifest as distinctly delineated lesions with an oval form. The MRI exhibits greater sensitivity in the detection of lipomas. Colonoscopy is an appropriate diagnostic procedure for the identification of the underlying etiology of intussusception. In addition, it has been suggested that the use of a colonoscopy procedure may serve to prevent the occurrence of unwarranted surgical interventions [4].

The present case exhibits a distinct nature due to the rarity of colo-colonic intussusception in the adult population. The uniqueness of our case lies in its distinctive leading point as well as the resolution achieved with the endoscopic use of an endoloop, thereby preventing the need for surgery, which is typically considered the ultimate therapy. Hence, the use of endoscopic intervention for the treatment of intussusception may serve as a viable therapeutic approach in some instances.

## Conclusions

Although intussusception is a common entity in the pediatric population, it is relatively rare in the adult population. Moreover, colonic lipoma as the leading entity is even rarer. This is an unusual case of a patient with classic symptoms of intussusception with a colonic lipoma as the etiology. The patient recovered well after the endoscopic treatment. This establishes endoscopic treatment as an upcoming modality for the same, which can be an effective alternative to surgery.
